# Adverse Drug Reactions in Pediatric Oncohematology: A Systematic Review

**DOI:** 10.3389/fphar.2021.777498

**Published:** 2022-02-03

**Authors:** Kristopher Amaro-Hosey, Immaculada Danés, Antònia Agustí

**Affiliations:** ^1^ Clinical Pharmacology Service, Hospital de la Santa Creu i Sant Pau, Barcelona, Spain; ^2^ Department of Pharmacology, Therapeutics and Toxicology, Universitat Autònoma de Barcelona, Barcelona, Spain; ^3^ Vall d’Hebron Research Institute, Barcelona, Spain; ^4^ Clinical Pharmacology Service, Vall d’Hebron University Hospital, Barcelona, Spain

**Keywords:** pharmacovigilance, adverse drug reactions, pediatrics, hematology, oncology, neoplasms, systematic review

## Abstract

**Introduction:** Adverse drug reactions (ADR) are an important cause of morbidity and mortality in pediatric patients. Due to the disease severity and chemotherapy safety profile, oncologic patients are at higher risk of ADR. However, there is little evidence on pharmacovigilance studies evaluating drug safety in this specific population.

**Methods:** In order to assess the incidence and characteristics of ADR in pediatric patients with oncohematogical diseases and the methodology used in the studies, a systematic review was carried out using both free search and a combination of MeSH terms. Data extraction and critical appraisal were performed independently using a predefined form.

**Results:** Fourteen studies were included, of which eight were prospective and half focused in inpatients. Sample size and study duration varied widely. Different methods of ADR identification were detected, used alone or combined. Causality and severity were assessed frequently, whereas preventability was lacking in most studies. ADR incidence varied between 14.4 and 67% in inpatients, and 19.6–68.1% in admissions, mainly in the form of hematological, gastrointestinal and skin toxicity. Between 11 and 16.4% ADR were considered severe, and preventability ranged from 0 to 74.5%.

**Conclusion:** ADR in oncohematology pediatric patients are frequent. A high variability in study design and results has been found. The use of methodological standards and preventability assessment should be reinforced in order to allow results comparison between studies and centers, and to detected areas of improvement.

**Systematic Review Registration:** https://www.crd.york.ac.uk/prospero/display_record.php?RecordID=96513, identifier CRD42018096513.

## Introduction

Adverse drug reactions (ADR) have been defined by the World Health Organization (WHO) as “a response to a drug which is noxious and unintended, and which occurs at doses normally used in man for the prophylaxis, diagnosis, or therapy of disease, or for modification of physiological function” (WHO, 1972).

ADR are an important cause of morbidity and mortality in patients of all ages, including pediatric population, and are considered a public health problem worldwide (Impicciatore et al., 2001; Clavenna and Bonati, 2009; Thiesen et al., 2013; Durrieu et al., 2014; Ramos et al., 2021). Children are more susceptible to ADR owing to insufficient standardized information, unlicensed and off-label use, unavailability of pediatric formulations, and physiological peculiarities inherent to age (Ramos et al., 2021).

Different systematic reviews and meta-analysis including ADR observational studies have found an incidence of ADR in pediatric inpatients ranging 0.6–16.8%, from 1.8 to 2.09% leading to hospital admission and 1.0–1.46% in outpatient setting (Impicciatore et al., 2001; Clavenna and Bonati, 2009; Smyth et al., 2012). In addition, ADR prevention in outpatients remains a public health and a patient safety challenge (Lombardi et al., 2018). A systematic review including 102 articles assessed preventability in only 19, which ranged from 7 to 98%. This high variability was explained due to a high heterogeneity in study designs, methods and settings (Smyth et al., 2012).

Risk factors for ADR in children are poorly characterized (Bellis et al., 2013; Lombardi et al., 2018). Age on admission, number of drugs, off-label drug use, and oncology diagnosis and treatment have been described as ADR risk factors (Bellis et al., 2013; Thiesen et al., 2013). Moreover, one of these studies stated the risk in oncology patients and found an increased risk for ADR (OR = 1.89 [95% CI 1.36–2.63]) (Thiesen et al., 2013).

Chemotherapy toxicity is a common cause of morbidity and mortality in most pediatric cancer patients, and a frequent cause of mid and long term sequel (Conyers et al., 2018). Even though drugs used in cancer diseases are described as a risk factor of ADR occurrence, and that ADR are frequent in oncology and hematology hospitalization wards, there are very few studies that have quantified or analyzed any of these aspects in pediatric population.

Oncohematological diseases have a high impact on children and their families, and on their quality of life. Improving the knowledge of ADR incidence, characteristics and preventability can be useful to compare results between studies and centers and to detect improvement areas, as a way to offer quality healthcare. Our aim was to perform a systematic review in order to describe the incidence and characteristics of ADR in pediatric oncology and hematology patients, to describe the methodology used in the included studies and, if possible, to identify preventive actions in order to minimize ADR occurrence.

## Methods

### Study Design

A systematic review of observational studies that evaluated the prevalence, incidence and/or characteristics of ADR in pediatric oncohematology was performed. This study was conducted in accordance with the recommendations of the Joanna Briggs Institute (Munn et al., 2015) for systematic reviews of observational epidemiologic studies that evaluate prevalence and incidence data, and the PRISMA recommendations for systematic reviews (Tricco et al., 2018). This study was registered (CRD42018096513) at PROSPERO systematic review database.

### Systematic Literature Search

A systematic literature search was carried out in PubMed from inception to 31st December 2020, both using free search and the combination of different MeSH terms (“Pediatrics,” “Neoplasms,” “Hematology,” “Antineoplastic agents,” “Drug-related side effects and adverse reactions,” “Iatrogenic disease,” “Prevention and control,” “Medical oncology,” and “Primary prevention”). References of the articles assessed for eligibility were also reviewed and included if considered relevant.

### Inclusion and Exclusion Criteria

Studies that described the incidence and/or characteristics of ADR in pediatric oncohematology patients or in pediatric population with a differentiated oncohematology subgroup were included in this systematic review. Articles describing infectious outbreaks related to immunosuppression, data from national or international clinical databases of spontaneous pharmacovigilance reporting systems and pharmacovigilance studies including one single drug or specific ADR were excluded. No language or other search filters were applied.

### Screening and Data Extraction

All articles were screened independently by two authors (KA-H, ID) to identify relevant studies based on titles and abstracts, and on full texts of potentially relevant papers if study relevance could not be determined from the titles and abstracts. For studies meeting inclusion criteria, data were extracted independently using a standardized data collection form defined and agreed previously. Data extracted included article identification, methodology characteristics (study design, setting, study aim, ADR definition and detection method, and causality, severity and preventability scales used), and relevant results (sample size, study duration, population characteristics, ADR frequency and description, severity and preventability). A third author (AA) participated in the review and in the data extraction in case of disagreement.

### Data Analysis and Quality Assessment

\This review focuses on both the incidence of ADR in a high-risk population and on the methodological characteristics of the studies included. Quality assessment was performed independently by two authors (KA-H, ID), using a scale designed and previously published (Laatikainen et al., 2017), available in the Supplementary Material. The scale includes six questions related to study design, study population, ADR definition and identification, causality assessment and result description. Each question can be evaluated as 0 or 1, where 0 indicates the poor quality of the study regarding that item. A third author (AA) participated in the critical appraisal in case of disagreement.

## Results

**FIGURE 1:**
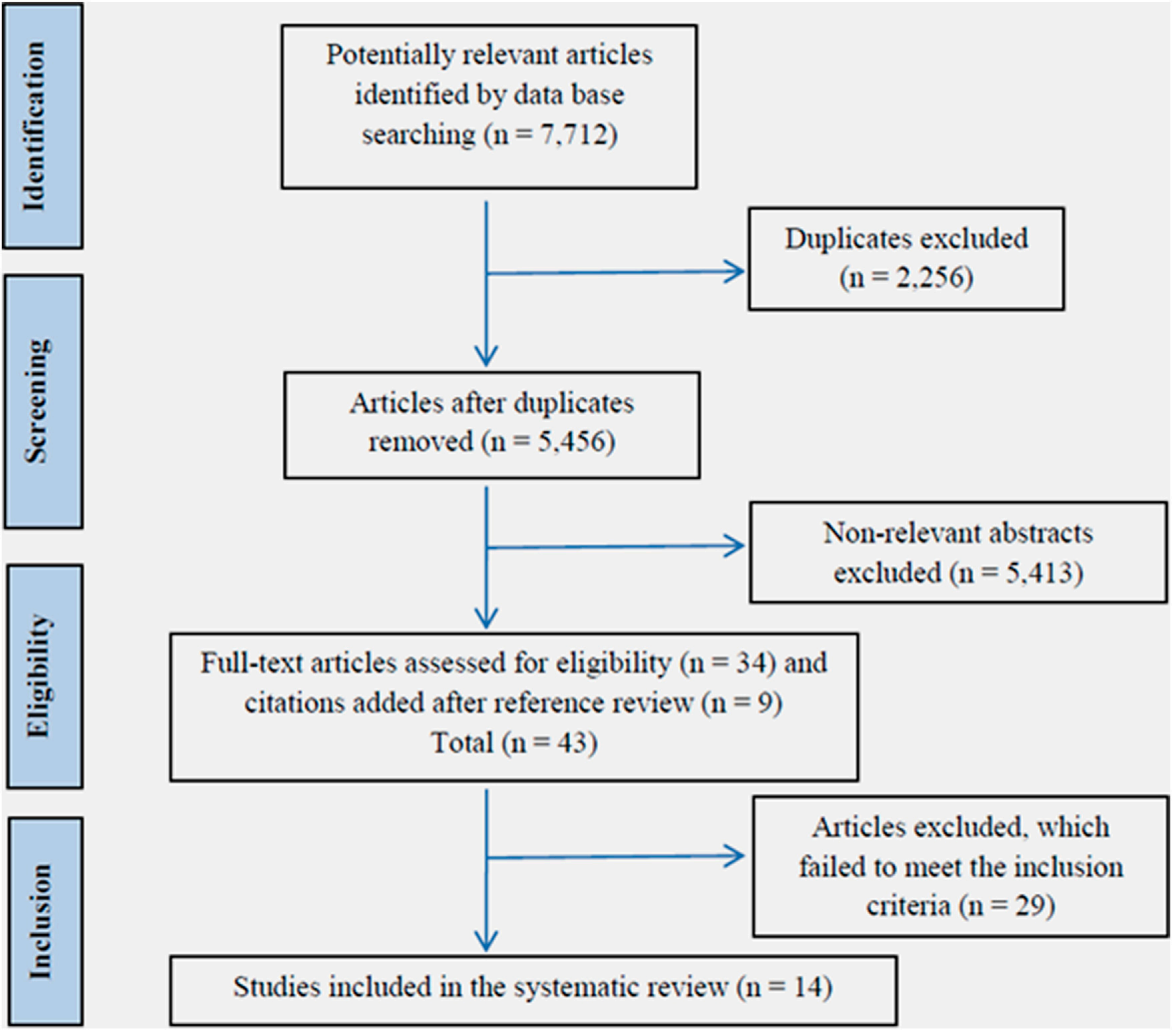
Study flow chart.

Using the research strategies defined previously, 7,712 studies were retrieved from PubMed database. Forty articles were considered relevant for eligibility and finally, considering inclusion and exclusion criteria, 14 studies were included in the systematic review (Collins et al., 1974; Mitchell et al., 1988; Queuille et al., 2001; Le et al., 2006; Gallagher et al., 2012; Posthumus et al., 2012; Barrett et al., 2013; Call et al., 2014; Langerová et al., 2014; Makiwane et al., 2019; Dittrich et al., 2020; Joseph et al., 2020; Morales-Ríos et al., 2020; Workalemahu et al., 2020). Due to the characteristics of studies found, a meta-analysis was considered not feasible to be carried out. Figure 1 shows the study flow chart.

Study characteristics and main results are summarized in Tables 1–4. Of the 14 studies included, six were carried out in pediatric oncology and hematology patients, and eight were carried out in general pediatrics and included a clear pediatric oncohematology subgroup. Four studies (Collins et al., 1974; Mitchell et al., 1988; Queuille et al., 2001; Le et al., 2006) were published before 2010, and 10 studies (Gallagher et al., 2012; Posthumus et al., 2012; Barrett et al., 2013; Call et al., 2014; Langerová et al., 2014; Makiwane et al., 2019; Dittrich et al., 2020; Joseph et al., 2020; Morales-Ríos et al., 2020; Workalemahu et al., 2020) were published later.

**TABLE 1:** Methodology characteristics in pediatric oncohematology studies.

References	Design	Setting	Main study aim	ADR definition	Detection method	Causality scale	Severity scale	Preventability scale
Barrett et al. (2013)	Retrospective	Inpatients	Assess the diversity of toxicities, the association with drug pairs and to compare the reported incidence of specific toxicities based on differences in dosing patterns	ADR (WHO)	Single centre pharmacovigilance database	NS	NCI CTCAE v4	Not evaluated
Call et al. (2014)	Retrospective	Inpatients	Investigate the effectiveness and efficiency of the use of a trigger tool for ADE detection	ADE	Triggers + chart review	NS	NCI CTCAE v4	NS
							NCC MERP MEI	
Collins et al. (1974)	Prospective	Inpatients	Assess the incidence and characteristics of ADR	ADR (WHO)	Intensive monitoring chart review + medical round + direct observation	NS	NS	Not evaluated
		Admission						
Joseph et al. (2019)	Prospective	Inpatients	Determine the incidence and characteristics of ADR, drug interactions and drugs involved	ADR (Edwards and Aronson)	Intensive monitoring chart review	Naranjo et al	Hartwig et al	Shumock and Thornton
Queuille et al. (2001)	Prospective	Inpatients	Evaluate ADE frequency and characteristics	ADE	Intensive monitoring chart review	Bégaud et al	EORTC tool	FAMC
							Hartwig et al	
Workalemahu et al. (2020)	Retrospective	Inpatients	Evaluate ADR associated to chemotherapy and related risk factors	ADR (WHO)	Chart review	WHO-UMC	Hartwig et al	Not evaluated

ADR, adverse drug reaction; ADE, adverse drug event; FAMC, factorial analysis of multiple correspondences; NCC MERP MEI, national coordinating council for medication error reporting and prevention medication error index; NCI CTCAE, National Cancer Institute common terminology criteria for adverse events; NS, not specified; WHO, World Health Organization; WHO-UMC, World Health Organization–Uppsala Monitoring Centre.

**TABLE 2:** Methodology characteristics in pediatric studies with oncohematology subpopulation.

References	Design	Setting	Main study aim	ADR definition	Detection method	Causality scale	Severity scale	Preventability scale
Dittrich et al. (2020)	Retrospective	Inpatients	Identify if ADR are adequately documented and reported to pharmacovigilance databases	ADR (WHO)	Chart review	WHO-UMC	NCI CTCAE v5.0	Not evaluated
Gallagher et al. (2012)	Prospective	Admission	Identify ADR causing admission in order to quantify and characterise them, and to determine their avoidability	ADR (Edwards and Aronson)	Intensive monitoring chart review	LCAT	Hartwig et al	Hallas et al
Langerová et al. (2014)	Prospective	Admission	Ascertain the incidence and characteristics of ADR related hospital admissions, and determine drug groups involved	ADR (Edwards and Aronson)	Intensive monitoring chart review	Naranjo et al	Not evaluated	Not evaluated
						LCAT		
						Edwards and Aronson		
Le et al. (2006)	Retrospective	Inpatients	Evaluate the incidence and common types of ADR in hospitalized children	ADR (WHO)	Spontaneous notification + triggers + medical round + drug monitoring	NS	Hartwig et al	Not evaluated
		Admission						
Makiwane et al. (2019)	Prospective	Inpatients	Describe the prevalence of ADR in pediatric inpatients	ADR (WHO)	Intensive monitoring chart review	Naranjo et al	Hartwig et al	Not evaluated
Mitchell et al. (1988)	Prospective	Admission	Provide information regarding pediatric hospital admissions prompted by ADR	ADR (NS)	Intensive monitoring chart review	NS	Not evaluated	Not evaluated
Morales-Ríos et al. (2020)	Retrospective	Inpatients	Estimate the frequency of ADR and their characteristics in hospitalized patients, as well as drugs related	ADR (WHO)	Single centre pharmacovigilance database	Naranjo et al	NOM-220-SSA1-2012	Not evaluated
		Admission						
Posthumus et al. (2012)	Prospective	Admission	Investigate the incidence and characteristics of hospital admissions related to ADR	ADR (Edwards and Aronson)	Triggers + chart review	Naranjo et al	Hartwig et al	Schumock and Thornton

Seriousness was evaluated in three studies using the following tools: ICH CIOMS definitions (Makiwane et al., 2019), ICH E2A guidelines (Dittrich et al., 2020) and NOM-220-SSA1-2012 guidelines (Morales-Ríos et al., 2020)

ADR, adverse drug reaction; ADE, adverse drug event; LCAT, Liverpool ADR, causality assessment tool; NCI CTCAE, National Cancer Institute common terminology criteria for adverse events; NS, not specified; WHO, World Health Organization; WHO-UMC, World Health Organization–Uppsala Monitoring Centre.

**TABLE 3:** Clinical results in pediatric oncohematology studies.

References	Sample size	Duration	Population characteristics	ADR frequency	ADR description	Severity (%)	Preventability (%)
Barrett et al. (2013)	1,713p	6.5 y	Age: 64% (2–12 y)	Incidence per year: 14.4–23.5%	Frequent ADR were neutropenia, increased ALT and febrile neutropenia (especially G3-4). The most toxic drug pair was methotrexate—vincristine. Twelve deaths were reported	NS	Not evaluated
			Gender: 53.1% male	326p, ADR NS			
			Dx: 52.1% leukemia, 28.5% neuroblastoma				
Call et al. (2014)	390p	4 y	Mean age: 11 y	Incidence: NS	Sodium polystyrene sulfonate and naloxone were the triggers most frequently related to an ADE	21 ADE (G3-4)	63.6%
			Gender: 55% male	Patients NS, 38 ADE			
			Dx: 54% leukemia, 24% solid tumor				
Collins et al. (1974)	63p	15 w	Mean age: 8.9 y	Incidence: 71% (45p/63p)	CT and antimicrobials were the drugs most frequently related to ADR. Gastrointestinal and hematologic ADR were the most frequently described during hospitalization. ADR most frequent during admission were nausea and vomiting with cyclophosphamide (9), cytosine arabinoside (6) and/or vincristine (6)	11% severe during hospitalization	Not evaluated
			Gender: 51% male	154 ADR: 63 (admission) and 91 (during hospitalization)			
			Dx: 44.4% leukemia				
Joseph et al. (2019)	176p	18 m	Age: 66.1% (2–12 y)	Incidence: 67% (118p/176p)	The most frequent ADR was rash (19), itching (10), anemia and gastrointestinal complaints (8). The most frequent drugs were vincristine (19) and methotrexate (16). Rashes were related to co-trimoxazole, allopurinol, dexamethasone, methotrexate and vincristine. Cases of itching were related to dexamethasone	16.4% severe	74.5%
			Gender: 55.9% female	131 ADR			
			Dx: 67.9% leukemia				
Queuille et al. (2001)	52p	50 d	NS	Prevalence: 65% (34p/52p)	Allergic reactions and medication errors were the most preventable ADE. CT was involved in >50% ADE	16% severe	53%
				155 ADE			
Workalemahu et al. (2020)	287p	25 m	Mean age: 7.1 y	Prevalence 41.5% (119p/287p)	Most frequent ADR were vomiting (16.3%), alopecia (15%) and febrile neutropenia (10.2%). Vincristine (85.4%), doxorubicin (61.7%) and cyclophosphamide (57.8%) were the most frequently prescribed drugs. Concomitant medication, etoposide, mercaptopurine, doxorubicin and >4 CT agents were identified as risk factors	74.1% moderate	Not evaluated
			Gender: 61.3% male	147 ADR			
			Dx: 23.3% leukemia, 22.6% Wilms tumor				

ADR, adverse drug reaction; ADE, adverse drug event; CT, chemotherapy; d, day; Dx, diagnosis; G3-4, grade 3–4; m, month; NS, not specified; p, patients; y, year.

**TABLE 4:** Clinical results in pediatric studies with oncohematology subpopulation.

References	Sample size	Duration	Population characteristics	ADR frequency	ADR description	Severity (%)	Preventability (%)
Dittrich et al. (2020)	T: 301p; POH: 31p	1 m	Median age (T): 5 y Gender (T): NS; Dx: NS	T: 26.9%; 81p; 132 ADR	All patients suffering multiple ADRs received CT. Cytostatics was the drug group most frequently associated to ADR (28.8%). Leukopenia and febrile neutropenia were the most common ADR	T: 12.1% G3-4 POH: NS	Not evaluated
				POH: % NS; p NS; 56 ADR			
Gallagher et al. (2012)	T: 6,821p; POH: 74p	1 y	Median age (T): 3 y and 1 m (IQR: 9 m, 9 y) Gender (T): 58.1% male; Dx: NS	T: 2.9% (240adm/8,345adm); 178p; 249 ADR	The most common ADRs were oncology related neutropenia (89), thrombocytopenia (55) and anemia (38); and immunosuppression (74) occurring in both oncology and non-oncology patients. The most frequent drugs were dexamethasone (68), vincristine (51) and doxorubicin (38). Oncology patients were much likely to have an ADR	T: 6.8% (≥G4) POH: NS	T: 22% POH: 6.7%
				POH: NS; 41p, 120 ADR			
Langerová et al. (2014)	T: 2.405p, 2,903adm POH: p NS, 143adm	9 m	Mean age (T): 7.1 ± 5.7 y; Gender (T): 57.1% male Dx: NS	T: 2.2% (64adm/2,903adm); p NS; ADR NS	The most frequent ADR were infections (16), febrile neutropenia (12) and mucositis (5). Cancer was described as a risk factor	Not evaluated	Not evaluated
				POH: 19.6% (28 adm/143 adm); p NS; ADR NS			
Le et al. (2006)	T: NS POH: NS	9y	Mean age (T): 7.0 ± 6.2 y; Gender (T): 52% male Dx: 31.5% had hematologic malignancies or disorders, or solid tumors	T: 1.6%/10 y (per year: 0.4–2.3%); 1,009p; 1,087 ADR	ADR with antibiotics were usually mild; anticonvulsants and CT were associated more commonly with severe reactions. Asparaginase was associated with 3% of ADR. One death in an oncohematological patient was reported	T: 11% POH: NS	Not evaluated
				POH: % NS; 318p; ADR NS			
Makiwane et al. (2019)	T: 282p POH: 23p	3 m	Median age (T): 1.4 y Gender (T): NS; Dx: NS	T: 18.4%; 52p; 61 ADR	ADRs were associated with CT (44.3%). ADR in POH included febrile neutropenia (6), anemia (4) and pancytopenia (3). Drugs related were doxorubicin, etoposide, vincristine, carboplatin and asparaginase. Oncology patients had an increased risk of an ADR	T: 11.5% POH: NS	Not evaluated
				POH: 56.5%; 13p; ADR NS			
Mitchell et al. (1988)	T: 10,297p POH: 725p	12 y	NS	T: 2.85%; 294p; ADR NS	The most frequent ADR in POH were neutropenia (41%), fever (38%) and leukopenia (29%). CT was involved in 94% of POH admissions. Three deaths were reported in oncohematological patients	Not evaluated	Not evaluated
				POH: 22%; 157p; ADR NS			
Morales-Ríos et al. (2020)	T: NS POH: NS	4 y	Age (T): 56.9% (2–11 y) Gender (T): 52% female; Dx (T): 72.2% neoplasms	T: 2.12–8.07% per year; 1,649p; 2,166 ADR	91.9% ADR led to admission and 94.5% required treatment in POH. Serious ADR were frequently related to antineoplastic drugs (81.2%), being febrile neutropenia (52.4%) the most common serious ADR. Cancer patient deaths were drug-related in 1.4% cases (febrile neutropenia commonly associated to death). Seventeen deaths were reported in oncohematological patients	T: 14.4% POH: 14.2%	Not evaluated
				POH: % NS; 1,190p; 1,494 ADR			
Posthumus et al. (2012)	T: 258p POH: 47p	5 m	Median age (T): 3 y and 6 m Gender (T): 56.6% male; Dx: NS	T: 18.2%; 47p; ADR NS	21 febrile neutropenia cases related to 20 different CT drugs (6 cases due to vincristine)	T: 0% POH: 0%	T: 13% POH: 0%
				POH: 68.1%; 32p; ADR NS			

Adm, admissions; ADR, adverse drug reaction; CT, chemotherapy; d, day; Dx, diagnosis; G3-4, grade 3–4; m, month; NS, not specified; p, patients; POH, pediatric oncohematology subgroup; T, total population; y, year.

### Methodological Results

Eight observational studies collected data prospectively, whereas six were performed retrospectively. Seven studies focused on hospitalized patients, four included admissions related to ADR and three analyzed both settings. No studies assessing outpatient setting were found. Twelve studies evaluated ADR, predominantly using WHO or Edward and Aronson definitions, and two studies used adverse drug events (ADE). Causality was estimated in nine studies, using mainly Naranjo and WHO-UMC scales. Severity was assessed in 11 studies, mostly using Hartwig et al. scale and NCI CTCAE criteria. Finally, preventability was only evaluated in five studies out of 14, using Shumock and Thorton in two of them. Ten studies used a single ADR detection method, and four studies used a combination of them: intensive monitoring chart review method was used in seven studies, chart review was used in four studies, and three studies based their results in triggers.

Critical appraisal is summarized in the supplementary material. Most of the studies defined adequately the study population and stated the causality assessment tool used (questions 2 [Q2] and 5 [Q5]). In contrast, results were considered not clearly described in half of the studies (Q6), as the information provided by the original articles on number of patients or ADR was missing. In addition, study design (Q1), ADR definition (Q3), and ADR detection method (Q4) were not clearly mentioned in three studies.

### Clinical Results

Sample size varied from 52 to 10,297 patients, as well as study duration, which ranged from 30 days up to 12 years. Age was expressed in means in five studies, as median in four or with percentage of patients in an age range (2–12 years old) in three; age values can be found in Tables 3, 4. Gender varied from 44.1 to 61.3% of males, and was not stated in four studies. Leukemia and solid tumors were the main cancer diagnosis, stated in seven studies. ADR incidence varied depending on study setting: it ranged from 14.4 to 67% in hospitalized patients, 19.6–68.1% in admissions caused by an ADR, and 2.12–71% in studies evaluating both settings. Chemotherapy toxicity described in the studies was related to hematological toxicity (anemia, febrile neutropenia), gastrointestinal toxicity (nausea, vomiting, transaminases increase), and skin (alopecia, rash). Both chemotherapy agents such as methotrexate, doxorubicin or vincristine, and antimicrobials were frequently related to ADR in oncohematology population. Severe ADR frequency described was 11–16.4%, and preventability also varied from 0 to 74.5%. Only four studies reported fatal cases, shown at the results tables.

Four studies included in this systematic review also assessed risk factors for an ADR. In general pediatric studies, Langerová et al. described oncology patients as an independent risk factor (OR = 9.8 [95% CI: 5.8–16.7]), as well as Makiwane et al. (OR = 7.3 [95% CI 3.0–18.9] and Gallagher et al., finding an even higher risk (OR = 29.7 [95% CI 17.4–50.9]). Workalemahu et al. described an increased risk for etoposide (OR = 1.99 [95% CI 0.93–4.27]), mercaptopurine (OR = 3.91 [95% CI 1.1–14.5), doxorubicin (OR = 2.32 [95% CI 1.3–4.2]) and >4 chemotherapy agents (OR = 2.7 [95% CI 1.5–4.7).

## Discussion

Even though ADR are an important cause of morbidity and mortality, are frequent in oncology and hematology, and chemotherapy is described as a risk factor, only 14 studies that assessed ADR were found. Incidence rates ranged from 14.4 to 61.3% in hospitalized patients and 19.6–68.1% in ADR leading to admission. A high heterogeneity in methodological aspects reviewed was also described and has likely influenced on the observed results. To our knowledge, this is the first systematic review on pharmacovigilance regarding pediatric oncology and hematology.

As mentioned previously, a high variability regarding methodology was found in almost every aspect of study design: sample size, study duration, study setting, population of interest, ADR detection method, the assessment of severity and preventability, and the different scales used. These findings could be explained by the different aim of each study, the effort to adapt the study to each local environment and available resources, research experience of the team and the moment in which they were designed and carried out, since methodology has evolved over time. These methodological differences have probably influenced on the clinical results found. A systematic review on ADR detection methods in hospitalized children was carried out (Ramos et al., 2021) and found that methods such as intensive monitoring chart review or trigger tools are more effective but time consuming, whereas spontaneous notification showed the lowest rate of detection. They concluded that most of the studies used a combination of methods, which might indicate a growing concern in ADR care in hospitalized children. This improvement in combined methods for ADR detection was previously suggested (Gonzalez-Gonzalez et al., 2013).

To our knowledge, there is no reference quality assessment tool for observational studies with other designs than cohort or case-control studies. A systematic review (Katrak et al., 2004) pointed out the variability in 121 published critical appraisal tools, regarding its intent, components and construction; this finding was later confirmed in another systematic review (Page et al., 2018), which concluded that there are several limitations of existing tools for assessing risk of reporting biases. STROBE statement (von Elm et al., 2007) or Johanna Briggs Institute (Munn et al., 2015) critical appraisal checklists are the most known tools, but their application was complex and troublesome. Therefore, the choice of the checklist used in this systematic review (Laatikainen et al., 2017) was agreed by the research team due to the lack of a standardized tool, its suitability to the type of studies included in the systematic review and to the aim of the critical appraisal analysis, and its easy application. The main area of improvement was the presentation of results, as results were insufficient or missing in half of the studies, and therefore it was considered to be the aspect most susceptible to introduce bias. Moreover, an adequate study design statement, ADR definition and identification clearly mentioned would likely reduce the risk of bias and improve study quality. Ten studies were published after the STROBE statement, which suggests a need to reinforce the use of these tools both during study design and manuscript drafting.

Incidence described in oncohematology pediatric patients was higher, in contrast with studies in pediatrics, which described an overall rate of ADR of 9.53 and 2.09% (hospitalized and admission, respectively) (Impicciatore et al., 2001). This finding is expectable and consistent with chemotherapy safety profile and ADR risk factors, such as cancer diagnosis or number of concomitant drugs. Moreover, it is likely that the use of different scales in causality and severity assessment has influenced on the results observed too.

Hematological, gastrointestinal and skin toxicities are the most frequently described ADR in the articles included, which are in tune with the expected safety profile of conventional chemotherapy. No studies with novel drugs such as monoclonal antibodies or tyrosine kinase inhibitors were found up to 2020. A recently published study (Amaro-Hosey et al., 2021) prospectively assessed drug safety with some specific therapies, including novel drugs and conventional chemotherapy. The most frequent ADR were hematological, infections and gastrointestinal. Incidence using days at risk was calculated regarding novel therapies: 1.1 and 5.3 ADR/100 days at risk for blood disorders and 0.8 and four ADR/100 days at risk for infections, related to pegaspargase and thioguanine respectively; and 0.6 ADR/100 days at risk for infections attributed to rituximab. Only four out of 14 studies included in the systematic review reported fatal cases, and the global incidence of fatal cases could not determined because the total population was not specified in two studies (Le et al., 2006; Morales-Ríos et al., 2020). This finding has been previously described and could either suggest that fatal ADR are very rare in children or are frequently underreported or not suspected (Bouvy et al., 2015).

ADR preventability is a key aspect to analyze, in order to identify areas of improvement to reduce ADR occurrence and improve patients’ life quality. A systematic review (Smyth et al., 2012) identified that preventability was only assessed in 19 out of 102 studies, and ranged from 7 to 98%. This finding is similar to result obtained in the current systematic review, which evidences that it’s an aspect poorly evaluated in pharmacovigilance studies and therefore should be encouraged.

This systematic review tries to add some evidence on an important health problem insufficiently studied that affects a fragile population. Summarized data on characteristics and incidence of ADR in this population is provided, as well as a methodological description in order to find areas of improvement. Defined inclusion and exclusion criteria, the selection of studies in pediatrics with an oncohematology subgroup, the lack of non-standardized critical appraisal tool that fitted the study characteristics and the use of a selected/concrete critical appraisal tool may have introduced bias, but was agreed and considered appropriate to enrich the results and the discussion. Great heterogeneity makes it difficult to compare results, but can also be interpreted as a need to establish methodology standards or to reinforce their use during study design and manuscript drafting, such as STROBE statement. Ultimately, our aim should be to provide a high quality research and healthcare to our patients and to improve their quality of life, regarding drug efficacy and safety.

In conclusion, ADR in oncohematology pediatric patients are more frequent than in general pediatric population, as expected. A high variability in study design and results has been found, which indicates a need to reinforce the use of methodological standards both in study design and manuscript drafting, in order to allow comparability between studies and to identify areas of prevention and improvement. Preventability assessment should be strongly encouraged in order to provide a high quality healthcare and to improve patients’ quality of life.

## Data Availability

The original contributions presented in the study are included in the article/Supplementary Material, further inquiries can be directed to the corresponding author.
