# Supplementary material for: Adverse Drug Reactions in Pediatric Oncohematology: A Systematic Review
Source: Front Pharmacol. 2022 Feb 3;12:777498. doi: 10.3389/fphar.2021.777498 (PMC8850382; doi:10.3389/fphar.2021.777498)
Supplement: Supplementary file 1 [file DataSheet1.docx]

Supplementary Material

**Table S1. Quality score (adapted from Laatikainen et al., 2017).**

| **Items** | **Quality score** |
| --- | --- |
| **Q1: Study design**  Prospective (real time data collection)  Retrospective (data extraction from inpatients treated in the past)  Unclear or poorly described | 1  1  0 |
| **Q2: Study population**  Study population is a comprehensive take of patients and eligible for study question  Patients population is biased toward specific variables and therefore provides unreliable answers to study questions | 1  0 |
| **Q3: ADR definition**  Definition similar to commonly used definitions (WHO, Edwards & Aronson)  No definition or definition deviating significantly from commonly used definitions | 1  0 |
| **Q4: Methods to identify ADR**  Explicitly described identification method by expert panel or existing standardized method  No description or poor description of method used | 1  0 |
| **Q5: Causality assessment**  Case-by-case causality assessment based on standardized criteria or expert review  No causality assessment for each individual case | 1  0 |
| **Q6: Results**  Frequency of studied ADR and the amount of patients included in the study clearly stated  No clear description of the amount of ADR or number of patients | 1  0 |

**Table S2. Critical appraisal of the studies included in this current systematic review.**

|  | | | | | | | |
| --- | --- | --- | --- | --- | --- | --- | --- |
|  |  | **Q1** | **Q2** | **Q3** | **Q4** | **Q5** | **Q6** |
| Pediatric oncohematology | Barrett et al., 2013 | 0 | 1 | 1 | 0 | 0 | 0 |
|  | Call et al., 2014 | 0 | 1 | 0 | 1 | 1 | 0 |
|  | Collins et al., 1974 | 1 | 1 | 1 | 1 | 1 | 1 |
|  | Joseph et al., 2019 | 1 | 1 | 1 | 1 | 1 | 1 |
|  | Queuille et al., 2001 | 0 | 1 | 0 | 0 | 1 | 1 |
|  | Workalemahu et al., 2020 | 1 | 1 | 1 | 1 | 1 | 1 |
| General pediatrics | Dittrich et al., 2020 | 1 | 1 | 1 | 1 | 1 | 1 |
|  | Gallagher et al., 2012 | 1 | 1 | 1 | 1 | 1 | 1 |
|  | Langerová et al., 2014 | 1 | 1 | 1 | 1 | 1 | 0 |
|  | Le et al., 2006 | 1 | 0 | 1 | 1 | 1 | 0 |
|  | Makiwane et al., 2019 | 1 | 1 | 1 | 0 | 1 | 1 |
|  | Mitchell et al., 1988 | 0 | 1 | 0 | 1 | 1 | 0 |
|  | Morales-Ríos et al., 2020 | 1 | 1 | 1 | 1 | 1 | 0 |
|  | Posthumus et al., 2012 | 1 | 1 | 1 | 1 | 1 | 0 |
